# News and Events of the Week

**Published:** 1911-01-14

**Authors:** 


### News and Events of the Week.

The Annual Ball in aid of the Funds of the Grantham Hospital was held at the Guildhall on Thursday, under the patronage of the Countess Brownlow, and the Annual Ball for the funds of the Victoria Nursing Association is to be held this (Friday), evening at the Guildhall.

---

The P. and O. Company will in future devote the steamers of their branch service to the conveyance of one class of passengers only to Australia *viâ* the Cape, at low fares ranging from £16 to £25. For this purpose the present fleet is being remodelled, and the two 11,000-ton steamers—the *Bendigo* and *Ballarat*—now in course of construction, will be arranged on similar lines. Full particulars will be supplied by the Manager, 3 and 5 East India Avenue, E.C.

---

The Secretary of State for India has sanctioned the construction of a medical college and hospital at Lucknow. The foundation stone was laid by the then Prince of Wales on his visit to Lucknow in December 1905, and the time since has been spent in elaborating and completing the full details of the project. The designs for the buildings are in the Indo-Saracenic style, the work of Colonel Sir Swinton Jacob, K.C.I.E. The college part of the scheme, the cost of which was met from private subscriptions, was commenced last year, and these buildings are now far advanced. The rest of the work will now be put in hand, and no effort spared to push the scheme to conclusion as rapidly as possible.

---

According to the *Paris Médicale*, a gentleman of Stockholm has prosecuted the Danish Anatomical Institute in the following circumstances : Twenty years ago the prosecutor had contracted to will his body to the Institute on consideration of a monetary payment. Since then, however, he has prospered and become rich, and now wishes to break the contract made in his days of misery. The Danish magistrates nevertheless declare that the contract is a licit one, that it is valid at law, and that it is binding and incapable of being annulled. The tribunal has moreover condemned the prosecutor to pay damages to the Institute of Anatomy for having broken one of the clauses of the contract in having had two teeth removed without first obtaining permission from the Institute.

---

We have received the reports for November 1910 of the Medical Officer of Health and of the Sanitary Inspector of the city of Aberdeen, according to which 323 births, 76 marriages, and 209 deaths occurred in the city during the month, giving rates per thousand respectively of 21.2, 5, and 13.7. Scarlet fever was the most prevalent zymotic, diphtheria came next, followed in order of prevalence by whooping cough and erysipelas. The numbers of cases respectively of these diseases were 51, 24, 18, and 16, and the deaths from each were 0, 2, 0, and 2. In addition, one case of typhoid fever, one case of puerperal fever, and one fatal case of cerebro-spinal meningitis were notified. Of non-notifiable zymotics, measles accounted for one case, phthisis caused 10 deaths, and other forms of tuberculosis 6 deaths. There were 3 fatal cases of influenza. The sanitary inspector made 493 inspections of food, resulting in 53 seizures of a total weight of 23,701 pounds. The health visitors made 56 visits to houses, finding 26 clean, 3 dirty, and 19 medium, and 8 improved on a second visit. Infant management accounted for visits to 222 infants newly born, of whom 193 were breast-fed.

Dr. James Hale, M.D., of Waterlake, Chiddingstone, Kent, who died on November 25 last, aged eighty-eight years, has left estate of the gross value of £20,080, with net personalty £12,207.

---

The Philosophic Faculty of the University of Marburg has conferred the degree of Doctor Honoris Causa upon Mr. Ernst Leitz, of Wetzlar, the chief of the well-known firm of microscope manufacturers.

---

Mr. Gideon de Gorrequer Griffith, L.R.C.P., M.R.C.S., of 34 St. George's Square, Belgravia, S.W., who died on November 15 last, left estate of the gross value of £21,754 6s. 9d., of which the net personalty has been sworn at £21,596 13s. 1d.

---

Dispensing by Medical Practitioners.—The Council of the Pharmaceutical Society has instructed Mr. W. S. Glyn-Jones, M.P., Parliamentary Secretary, to urge the Privy Council to authorise an investigation into the conditions under which the storage, compounding, and dispensing of medicines and their distribution are carried on in various surgeries, dispensaries, and similar establishments in Great Britain.

---

The Council of the Pharmaceutical Society having considered the recent Blue-book on unqualified medical practice, has resolved to call the attention of the Privy Council to the fact that at present the precautionary measures imposed by statute upon chemists and druggists do not apply to medical practitioners. The Council further desires that the Privy Council should investigate the conditions under which the dispensing of medicines is carried on in various surgeries, dispensaries, and similar establishments in Great Britain.

---

Dr. Labadie-Lagrave, a French medical hero, has just been decorated at the age of sixty-six. He was a house-surgeon in 1867 and became a hospital physician in 1879, and is a distinguished gynæcologist. "He has taken," states the *Figaro*, with evident reflection on the tardiness of conferring the honour, "not less than thirty-nine years to pass from the grade of Chevalier of the Legion of Honour to that of officer. He had the red ribbon given him in 1871 for a deed in battle. In his book, 'The Invasion,' Ludovic Halévy has recounted how a young man took the lead at the risk of his life and saved an important convoy of ammunition and provisions from Prussian ambushes. The hero of that deed of arms is Dr. Labadie-Lagrave, chevalier of 1871 and officer of 1911."

---

A Holiday in the Canaries.—In these days of frost and sleet the vision of palm-trees, blue skies, and golden sunshine is to the average practitioner merely a vision, the enjoyment of which depends largely on his imaginative capacity. And yet a winter holiday in the Canaries is now within nearly everybody's reach, thanks largely to the splendid efforts of the Yeoward Line of steamers. For ten guineas the passenger by one of their boats obtains a twenty-three days' cruise, which, as we know from experience, can be one of the most enjoyable of ocean trips. Yeoward Brothers, whose address is 27 and 29 Stanley Street, Liverpool, issue a most attractive illustrated booklet giving full particulars about the trips. We shall be glad to know more about the new steamers, and to hear from medical men who have achieved the tour—and climbed the Peak !

